# Heuristic Evaluation of Ehealth Interventions: Establishing Standards That Relate to the Therapeutic Process Perspective

**DOI:** 10.2196/mental.4563

**Published:** 2016-01-13

**Authors:** Amit Baumel, Fred Muench

**Affiliations:** ^1^ The Feinstein Institute for Medical Research Department of Psychiatry North Shore–LIJ Health System Glen Oaks, NY United States; ^2^ Hofstra North Shore LIJ School of Medicine Hempstead, NY United States; ^3^ The Feinstein Institute for Medical Research Department of Psychiatry North Shore–LIJ Health System Great Neck, NY United States

**Keywords:** eHealth, mHealth, digital health, mobile health, heuristics, evaluation, principles, therapeutic process

## Abstract

In recent years, the number of available eHealth interventions aimed at treating behavioral and mental health challenges has been growing. From the perspective of health care providers, there is a need for eHealth interventions to be evaluated prior to clinical trials and for the limited resources allocated to empirical research to be invested in the most promising products. Following a literature review, a gap was found in the availability of eHealth interventions evaluation principles related to the patient experience of the therapeutic process. This paper introduces principles and concepts for the evaluation of eHealth interventions developed as a first step in a process to outline general evaluation guidelines that relate to the clinical context from health care providers’ perspective. Our approach was to conduct a review of literature that relates to the examination of eHealth interventions. We identified the literature that was most relevant to our study and used it to define guidelines that relate to the clinical context. We then compiled a list of heuristics we found to be useful for the evaluation of eHealth intervention products’ suitability for empirical examination. Four heuristics were identified with respect to the therapeutic process: (1) the product’s ease of use (ie, usability), (2) the eHealth intervention’s compatibility with the clinical setting, (3) the presence of tools that make it easier for the user to engage in therapeutic activities, and (4) the provision of a feasible therapeutic pathway to growth. We then used this set of heuristics to conduct a detailed examination of MyFitnessPal. This line of work could help to set the bar higher for product developers and to inform health care providers about preferred eHealth intervention designs.

## Introduction

In recent years, the number of available eHealth interventions aimed at treating behavioral and mental health challenges has been growing. Tens of thousands of health, wellness, and medical applications are now available for download from online stores [1], and it is clear that eHealth interventions will play a substantial role in shaping health care in the future [2].

Scholars have described the need to empirically evaluate the efficacy of eHealth interventions, to develop standards of assessment [3], and to find new ways of evaluating eHealth interventions as they evolve [4]. From the perspective of health care providers, the state-of-the-art evaluation of eHealth interventions is expensive, time-consuming, and involves a rigorous process of validation, primarily in terms of clinical aspects, data security, and legal agreements. While it is clear that not all of the eHealth interventions that vendors propose to health care providers can be empirically examined, it is important to develop assessment methods for each new product [4]. In effect, eHealth interventions should be examined prior to clinical trials so that the limited resources for empirical research can be invested in the most promising products.

In reviewing the literature, we found a gap in terms of the minimum standards that eHealth interventions targeting behavioral and mental health should meet with regard to patients needs in the therapeutic process prior to empirical examination. This paper discusses relevant principles and concepts for evaluating eHealth interventions from this perspective, and outlines an approach to screen and identify suitable products. In the following sections, we outline the literature most relevant to our study, focusing on models informing eHealth interventions design, eHealth interventions’ heuristic evaluation and rating systems.

### Learning from Models of eHealth Interventions

There are several models that have informed the development of eHealth interventions [5-9] and established the fundamental principles of the design process. These models provide eHealth interventions developers with ways to transform clinical understanding and theories into actionable product designs aimed at creating behavioral change.

Fogg’s model [8] relates to the performance of a specific target behavior and focuses on the dynamic between the user’s motivations, abilities, and triggers. According to Fogg, for a person to perform a target behavior, he or she must be sufficiently motivated, have the ability to perform the behavior, and be triggered to perform the behavior. When these factors occur at the same time and exceed a certain threshold, the target behavior will be performed by the user. Fogg provides an example of target behavior, namely having website users provide their email address in order to receive a newsletter. Users may see the request to type in their email (ie, trigger) and find it easy to type the email address (ie, ability). However, if they have no motivation to do so the target behavior will not occur. It seems that if users have much more motivation to receive this newsletter they might be willing to do much more than to just type their email address. While Fogg’s model does not provide a framework for evaluating more complex target behaviors or treatment outcomes, it does provide a point of reference for evaluating a feature’s ability to create a specific behavioral change.

Mohr et al’s model [5] is a detailed behavioral intervention technology (BIT) design model. It provides a step-by-step conceptual and technological framework that ensures the product is designed to be both useful and usable, while keeping in mind the clinical aim. The model is able to provide clear definitions and establish a common language for all parties involved in eHealth intervention development. For example, the model defines 5 questions that developers should answer when designing BITs or eHealth interventions: (1) “why,” the clinical aim; (2) “conceptual how,” the behavior change strategies used; (3) “what,” the technical elements of the intervention (eg, notifications); (4) “technical how,” the characteristics of the various elements (eg, medium, personalization); and (5) “when,” the time when the intervention should be delivered.

These models emphasize the importance of relating the product design to the clinical context—a factor that we find to be particularly relevant in the mental and behavioral health domains. At the same time, these models view the product from the developer’s perspective, which makes it difficult for a person external to the development process to evaluate the product without having to trace the developer’s and designer’s intentions. In light of this drawback, we suggest that health care providers can use a global approach for evaluating a product as a whole, while taking the clinical context into account.

### Heuristic Evaluation of eHealth Interventions

In contrast to these eHealth intervention development frameworks, heuristic evaluation is a method that has been broadly researched and used for assessing eHealth and technology products, particularly in terms of identifying problems with user interface usability [10]. Heuristics are broad principles of product design that can be inspected by evaluators prior to empirical testing. Heuristic evaluations can be implemented widely and transferred easily to new organizational contexts [11]. The advantage of heuristic evaluation is that it enables the cost-efficient identification of design problems [12], which is valuable in situations where time or budgetary resources are limited [11].

Kientz and colleagues [13] developed a set of 10 heuristics intended to find design problems in persuasive technologies aimed at health behavior change. Kientz’s heuristics relate not only to user interface usability (eg, “appropriate functionality,” “usable design”), but also to some aspects of the design that engage the user in the therapeutic process such as “use of positive motivation strategies.” Kientz et al compared the performance of these heuristics to that of Nielsen’s heuristics [14] and demonstrated the effectiveness of their heuristics in uncovering the main design problems in the domain of persuasive health technologies. Kientz’s heuristics reveal the need to take the user’s emotional perspective into account when evaluating the product’s ability to change user behavior.

App rating systems can also inform the heuristic evaluation of eHealth interventions, since the subscales reflect the scholarly understanding of relevant issues. PsyberGuide [15] is a system through which experts from the clinical and research field can rate mental health apps and software. The rating scale consists of subscales based on the extent of empirical research and support associated with the product. Another rating system was introduced by the Anxiety and Depression Association of America for rating anxiety-related apps [16,17]. The rating scale includes dimensions such as ease of use (ie, usability), personalization, and empirical evidence. Most recently, Stoyanov et al [18] developed the Mobile Apps Rating Scale (MARS) for health and well-being based on the quality rating criteria found in the research literature. MARS provides a detailed framework for rating apps according to their engagement, functionality, aesthetics, information quality, and subjective quality. Another notable initiative in this domain is JMIR mHealth peer-review tool for mobile apps. JMIR mHealth aims to build a database of peer-reviewed and evaluated apps and then to use this data to identify important domains within the evaluation of mobile health applications [19].

This stream of literature highlights the need to develop a greater understanding of the core components of eHealth interventions and provides a framework for researchers to discuss and compare different products. While these efforts have been advancing the science rapidly, there has been little focus on establishing principles for product evaluation in terms of the clinical context and the patient’s experience of the therapeutic process.

Issues related to the therapeutic process have often been overlooked in the evaluation process because they are thought to be inherent in eHealth intervention design models and application development. However, being able to evaluate eHealth interventions targeting behavioral and mental health in terms of the patient’s needs within the therapeutic process could contribute to the establishment of standards that eHealth interventions would have to meet prior to empirical examination. Below, we review the core evaluation principles that we found to be helpful in understanding the potential of eHealth interventions with regard to the clinical context. The primary aim of this paper is to describe these general principles for evaluating eHealth interventions as a first step within a process that demands rigorous testing of these heuristics across a number of contexts, using multiple eHealth products.

### Process for Defining Heuristics

The initial process for defining the most relevant heuristics was composed of several steps. First, the study authors defined an overarching framework for defining heuristics. Second, the authors reviewed covered literature, heuristics, and rating scales within the behavioral health domain. Within the overarching framework, they discussed whether an evaluation principle that corresponds with the patient’s clinical context is missing and can contribute to the evaluation process of new technologies (see Textbox 1 for a list of reviewed heuristics, principles, and authors’ comments). Then a group of 4 scholars was gathered. The group consisted of 3 psychologists (including the study co-authors) with experience in user-centered design and evaluation and 1 psychiatrist with experience in managing both health care clinics and research projects on technology-assisted interventions in behavioral health. The group reviewed and discussed the gathered principles, made modifications, and combined similar principles until reaching consensus. Finally, the authors provided a detailed examination of the MyFitnessPal app using the set of articulated heuristics. In the subsequent section, we outline our overarching framework articulated for defining heuristics.

### Overarching Framework for Defining Heuristics

#### Evaluating the Potential Success of eHealth Interventions from the Therapeutic Perspective

This paper relates to the context of the patient’s needs from a clinical point of view. While there is overlap between the literature in several areas (eg, usability), we have attempted to explain these overlapping concepts within the context of the therapeutic process. Accordingly, we have found it useful to treat the relation between the product’s usability and its therapeutic potential as the relation between a measure’s reliability and its validity [20], wherein usability is compared to reliability and therapeutic potential to validity. In effect, a product can be usable without exhibiting any therapeutic potential, but it cannot have therapeutic potential without being usable. On the same note, we relate to heuristics and principles reviewed within the study scope (Textbox 1) as a starting point for the process of evaluating the therapeutic potential, whereas a product that does not answer, for example, safety or quality of information concerns [21] cannot hold a strong therapeutic potential.

#### Examining the Product as a Whole

One of the desired outcomes of heuristic evaluation is to be able to examine products in a short amount of time, and in a way that is easy to communicate and transfer to others [11]. Therefore, it is important to examine the product as a whole, rather than breaking it down into smaller pieces. Indeed, understanding the gestalt of eHealth interventions is rarely discussed in the literature; prior research has tried to separate component parts according to their therapeutic mechanisms, instead of evaluating the phenomenological experience of using the product. Similarly, since many eHealth interventions attempt to address complex problems, such as depression, self-management of chronic illnesses, and addiction [5], we recognize that there must be room for creativity in product design. Such creativity can only be engaged, however, when the heuristics reflect broader principles for product evaluation.

**List of established Heuristics/Principles based on covered literature.**

Comments regarding missing evaluation principles are provided in the section following the textbox. The same heuristic/principle may appear under more than 1 subject.

1. Usability/Ease of Use/Functionality

- Visibility of system status
- Match between system and the real world
- User control and freedom
- Consistency and standards
- Error prevention
- Recognition rather than recall
- Burden and effort reduction
- Flexibility and efficiency of use
- Aesthetic and minimalist design
- Help users recognize, diagnose, and recover from errors
- Help and documentation
- Not irritating or embarrassing (eg, the technology should not inaccurately record or misrepresent the user’s behavior)
- Appropriate functionality
- Appropriate time and place of information, feedback, and assistance
- Easy to use

2. Aesthetics

- Aesthetically appealing design
- Appropriate design for the target audience

3. Safety

- User’s privacy is appropriately protected
- Data is secured
- The content is based on evidence-based principles and provides reliable information

4. Content

- The content is clear, logical, and correct
- The content is based on evidence-based principles and provides reliable information (eg, based on behavioral activation, etc)
- The content provides the tools or methods to accomplish its purpose
- The extent of content covered is comprehensive but concise
- The content is tailored

5. Engagement

- Entertaining
- Interesting
- Customized/tailored
- Interactive
- Relevant to target audience

6. Persuasive Design

- Motivate
- Educate users about the connection between user actions and desired outcomes: While the connection between user actions and desired outcomes is stated, the relation between this connection and the adherence to the therapeutic process should also be explicitly stated when examining the product within the clinical context.
- Sufficient motivation and triggers to promote desired behaviors

7. Research Evidence (this information is not gained from direct examination of the product)

- Data from pilots, open studies, and randomized controlled trials
- The credibility of the organization that administered the research

8. Owners’ Credibility (this information is not gained from direct examination of the product)

- The app comes from a legitimate source
- Product has an advisory board with clinical-thought leader input

The usability principle (“Usability/Ease of Use/Functionality” in Textbox 1) should relate to the evolving nature of the user’s expectation as technology evolves all the time. From the user perspective, is there a benchmark to understand their evolving expectations regarding product usability? This benchmark could support the reviewer in the understanding of the patient’s changing expectations. If the relation between the technology and the clinical context in which it is being used is not clearly stated, it may affect the user needs in terms of content covered, reasons to use it, and impact the product’s applicability to be perceived as engaging and persuasive (see “Content,” “Engagement,” and “Persuasive Design” in Textbox 1). While relating to the question of meeting the product’s clinical aim, using different heuristics to examine content, engagement, and persuasive design may miss the gestalt of the intervention. It can be related to, after examining all other heuristics, by asking whether the product provides a strong case for reaching the desired clinical aim (see “Content,” “Engagement,” and “Persuasive Design” in Textbox 1). The direct impact of products in promoting desired therapeutic activities by making it “easier” to conduct them (ie, lowering the investment required for conducting these activities) should be explicitly stated and evaluated (see “Persuasive Design” in Textbox 1).

#### Design That Nurtures Users’ Motivation

We assume that the user’s motivation to engage with the computer program and to comply with the intervention increases the chances of achieving behavioral change, especially when the product concentrates on behaviors the user finds difficult [8]. User motivation can also help in eHealth program adherence, even when the program is not tailored to meet the user’s needs. Therefore, all of the heuristics we list below consider how design can nurture the user’s motivation by relating the product’s ability to provide a suitable user experience to the user’s therapeutic needs, beliefs, desires, and intentions.

### Definitions

Features: Tools and components of the eHealth interventions that are being used to deliver the intervention (eg, assessments and psychoeducation ingredients).

Product usability: The extent to which the product is easy for the average user to learn and to use.

### Heuristics

#### The Product Should Be as Easy to Use as Products in Similar Settings.

The product usability and ease of use have been described as a significant factor within the evaluation process in the covered literature [11,13,18]. Kientz and colleagues [13], for example, related appropriate functionality to the product, stating that: “The technology should function effectively in the user’s environment by being easy to use and integrate into one’s daily life and routine.” From the provider’s perspective, we focus on the user’s needs and expectations, which are based on available products in similar settings. We believe that users’ expectations are mostly general and not defined by the product’s domain (eg, entertainment, education, social media). In effect, products that are in general use set the user’s expectations of other similar products.

**Figure 1:**
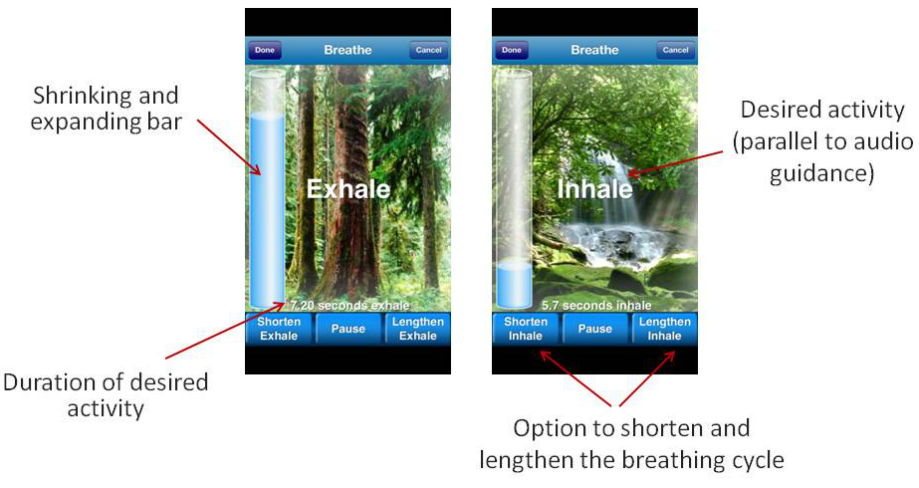
Breathe2Relax screenshots.

As an example, a tool providing breathing exercises (as part of a stress reduction feature) that consists of a text message explaining a breathing technique and asking users to do this exercise will probably result in poor cooperation. A breathing tool (Figure 1) that consists of (1) a bar that shrinks and expands, (2) an audio-recorded voice that directs the user throughout the exercise, and (3) the ability to adjust the time of the breathing cycle (ie, different people have different breathing cycles) will probably result in a higher level of user engagement.

#### The eHealth Intervention Should Respond to the User’s Needs with Respect to the Specific Clinical Setting

While fundamental principles of eHealth interventions’ design process explicitly relate to the clinical setting by addressing the clinical aim [5] and the user’s current mental state [8], it seems that sets of heuristic evaluation and rating systems [13,15,18] do not explicitly relate to the user’s needs as a derivative of the clinical setting. This gap can be filled by relating within the evaluation process to the implication of the clinical setting on the user’s therapeutic needs.

Below, we introduce as examples basic principles derived from this perspective for a product meant to be used in 2 different clinical settings: without a clinician support (ie, standalone) or to complement the clinician’s work (ie, as a supplement to therapy).

Standalone: As the user has to work through the therapeutic process alone without external guidance, it seems that he or she might benefit from:

1. A workflow that is tunneled, simple to understand, and includes tutorials, where applicable;
2. Ways to nurture and reinforce their inner motivation and to adapt over time to fluctuating and changing motivations;
3. The ability to receive relevant referral for external resources when needed.

A supplement to therapy: From the provider’s perspective, a product can be easily integrated and used as a supplement to therapy when it meets the clinician’s standards by providing tools, exercises, and psychoeducation in line with the clinician’s practice. The clinician should also have the flexibility to assign different product features based on the patient’s current state and be able to receive reports on the patient’s engagement with the program.

#### The eHealth Intervention Should Make it Easier for Users to Engage in Therapeutic Activities by Providing Them with the Relevant Tools “In House”

EHealth interventions are part of a therapeutic intervention. As such, they often include recommendations to engage in activities for a therapeutic gain. Fogg [8] suggests that the lower the level of investment necessary for carrying out desired activities, the higher the chances for these activities to occur. This notion is congruent with studies in the behavioral health domain demonstrating that availability and accessibility of services promotes their utilization [22-27]. We thus suggest that eHealth interventions should also be evaluated by their ability to promote desired behaviors by providing tools that decrease the investment needed for these behaviors to occur.

Some examples from the mental and behavioral health domains are:

1. A suggestion to socially engage with other people might benefit from including features such as: (1) in-house engagement options (eg, a click button that makes it easier to send an email or make a call); (2) reminders to perform the desired activity, if applicable (eg, automatic pop-up reminders on the mobile screen); and/or (3) a list of modeled narratives to choose from and customize.
2. A cognitive behavioral treatment (CBT) app that promotes the documentation of unhealthy thoughts might benefit from providing the user with a documentation tool.

#### The eHealth Interventions Should Provide a Feasible Therapeutic Pathway to Growth

Relating to the product’s clinical aim and how it is met is a crucial part of the eHealth product design process [5,6,8] and evaluation processes of eHealth interventions. Stoyanov et al [18] examine whether the mobile app has specific, measurable, and achievable goals, and whether the content is relevant to meet the app’s goals. Kientz and colleagues [13] provide a notable exception relating to the user motivation and experience in this process stating that: “users should understand why the actions they do promote positive behaviors and how their goals are being met.”

From a clinician perspective, we suggest addressing these principles under one umbrella through the concept of a feasible therapeutic pathway to growth. Within the evaluation process, this concept is meant to capture whether the product features are built in a way that helps the product meet the eHealth intervention goal from the perspectives of both health care providers and patients:

1. The eHealth intervention features should reasonably help the user meet the therapeutic goals. Scholars within the relevant domain should be able to roughly evaluate (based on experience and knowledge of evidence-based interventions) whether the product’s features are sufficient enough to meet its goals.
2. In order to benefit users’ adherence with the therapeutic process, the technology should engage users by providing a connection between their actions and therapeutic goals. This might be accomplished if the features are integrated in a way that helps the user to understand his or her current state and how to use each feature on the pathway to growth.

### Evaluation Process

**Figure 2:**
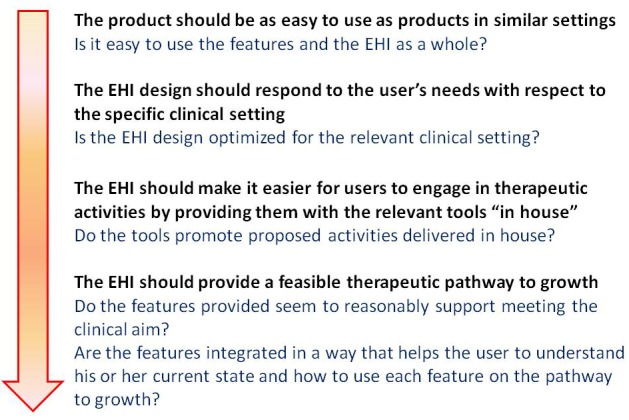
Heuristic evaluation process.

The heuristics have been presented in the order we believe is most useful for examining eHealth interventions (Figure 2). The first question is whether the product “looks and feels” easy to use (ie, is usable) in comparison to widely used products. When examining the product’s usability, the evaluator learns about the product and can consider the extent to which it corresponds with the clinical setting. These considerations allow the evaluator to then look closer at the features, assessing whether they are engaging and whether the necessary tools accompany the features “in house.” One might argue that since the “feasible therapeutic pathway to growth” heuristic is more general than the preceding one, it should be examined earlier. However, we have found it more useful to examine the “feasible therapeutic pathway to growth” heuristic only after examining all of the product’s features and becoming familiar with them.

### MyFitnessPal as an Example

To further elaborate on each heuristic, we use the example of the MyFitnessPal (MFP) app previously examined by Mohr et al [5]. MFP is a mobile phone and computer application that is free to download; is available for iOS, Android, and Windows operating systems; and used by more than 40 million users [28]. The clinical goal of MFP is to promote weight loss by reducing caloric intake and increasing physical activity.

Since the aim of this paper is to provide guidance and clear definitions regarding the relevant heuristics, we address each heuristic in reviewing the app, a process we conducted in March 2015. For practical purposes, we focused on the app’s ability to promote weight loss only in terms of reducing caloric intake.

#### The Product Should Be as Easy to Use as Products in Similar Settings

**Figure 3:**
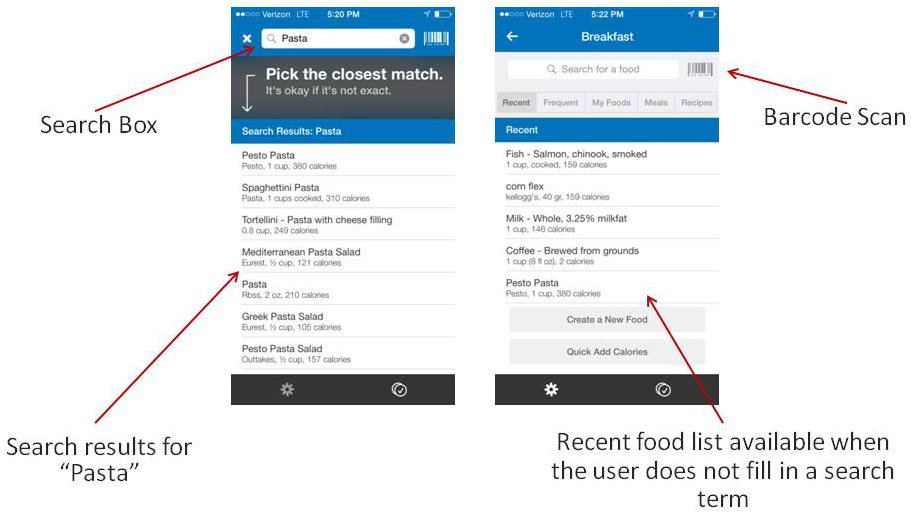
MyFitnessPal food-picking screenshots.

The user’s main investment in the MFP program is to provide information about food consumed each day. The average user of similar apps is used to utilizing search boxes to find the relevant food in the app’s database. Many search boxes in other programs also provide a word completion option, making it easier to find the relevant searched-for item. The MFP app uses several components that meet the average user’s expectations when it comes to adding the relevant food (Figure 3), including (1) a search box that enables the user to find products related to the search term (eg, the term “pasta” provides several common pasta dish options), (2) an option to choose items from a “recent foods” list composed of previously reported foods, and (3) an option to use a word-completion function (this function is device-dependent; applicable for the iOS version of the app at the time of examination). Another available way to find the caloric and nutritional information for food consumed is scanning the product’s bar code into the app and clicking the relevant button on the mobile screen. While it would not be considered as a standard function by users of all ages, we believe the bar-code scanner meets the expectations of young and tech-savvy users. Based on these functions, it seems that the MFP app looks and feels easy to use (ie, usable) and offers features that meet the standards of the average user.

#### The eHealth Interventions Design Should Respond to the User’s Needs with Respect to the Specific Clinical Setting

While the MFP app is built to be used as a standalone product, probably by a subclinical population, we would like to consider its applicability as a supplement to treatment for the purposes of this study. While this app doesn’t seem to be designed to replace clinicians’ work with diagnosed patients, there are certain features in the app that make it helpful as a supplement to treatment that we would like to address. A certified dietitian can recommend that a patient use the app in order to easily track the patient’s caloric intake. In this case, the app would become a smart workbook, helping the patient to adhere to “the calories watch” and would enable the dietitian to easily view past caloric intake by examining the patient’s diary. While MFP can offer a path of communication between the patient and the dietitian through the “friend” feature (ie, the patient adds the dietitian as a friend in the app), it is not designed to meet this need. For this product to be optimized as a supplement to therapy, its design should include ongoing automated reports and alerts sent to a designated dietitian and provide established communication pathways between the dietitian and the patient (including a feature that enables the dietitian to set goals and rewards tailored to the patient). These features also add an element of supportive accountability [29], which is especially important with higher-burden behaviors, such as food logging, and any reactive data entry by the end user.

#### The eHealth Intervention Should Make it Easier for Users to Engage in Therapeutic Activities by Providing Them with the Relevant Tools “In House”

Generally, the program design provides the relevant tools to carry out almost every activity it recommends and to minimize the investment needed from the user. MFP provides the user with the ability to easily find the nutritional value of each food and to receive summary reports. It enables the user to easily share accomplishments with friends using Facebook, phone contacts, or email. It provides live community forums and groups that users can join, which are managed within the application. The program also provides healthy recipes with a designated space to save the recipes.

#### The eHealth Intervention Should Provide a Feasible Therapeutic Pathway to Growth

The app includes many relevant features to promote weight loss by reducing caloric intake. The main feature is the feedback the user receives, which provides a clear call for action by indicating how many calories the user can consume for the rest of the day. Other features include nutritional summaries, recipe collections, community (eg, blogs and newsfeeds sharing ideas and thoughts about healthy living), goal setting, and social engagement. In light of these features, it seems reasonable that the product enables users to watch their diet and adjust their caloric intake.

**Figure 4:**
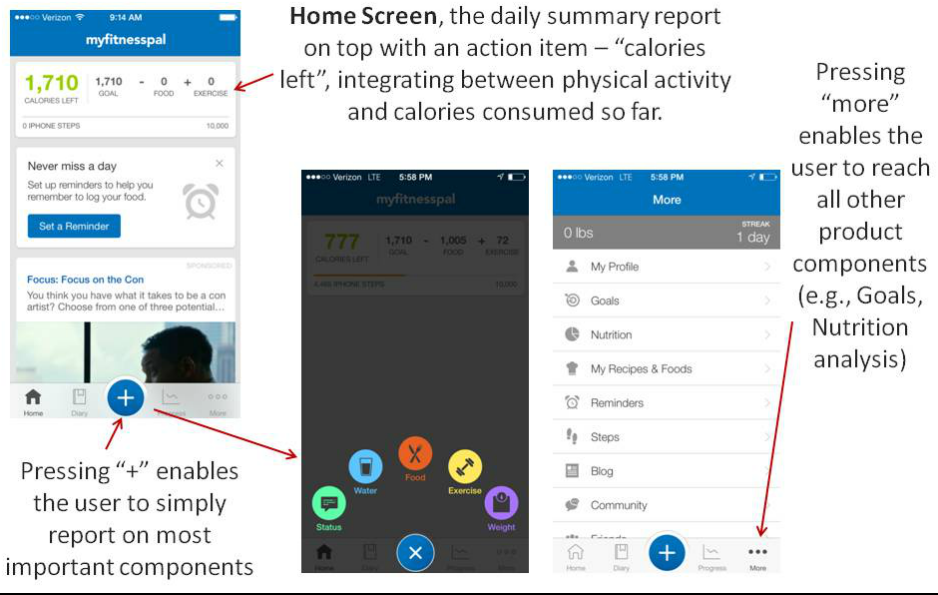
Navigation between MyFitnessPal features.

While the product provides most of its features as separate tools and does not offer comprehensive guidance as to how and when to use them, there are several elements that make it clear for the user. Certain features in the product seem to be more important and therefore receive a more prominent spot in the product’s graphical user interface. The main features are the calorie consumption assessment and summary report presented on the main page (Figure 4). In addition, there are automatic reminders prompting the user to complete the assessment. Data components that need to be collected (eg, food, water) throughout the day are accessible by clicking the “+” button at the bottom of the screen, while pressing the “more” button reveals all other features.

This approach relies on several factors. First, the most important features are the calorie consumption assessments and the accompanying report. Second, since the informed user may do many different things to maintain or change a certain diet, the app does not offer only 1 way to use the tools provided; rather, it keeps all options open. Third, from the user’s perspective, the pathway to growth is very clear: it depends on the user’s ability to meet the set caloric intake goal (in relation to physical activity). The daily feedback reflects the user’s goal and pathway to growth by providing the amount of calories he or she can consume for the rest of the day.

## Discussion

In this paper, we have described heuristic evaluation principles and how they correspond to the patient’s clinical context in order to outline an approach for identifying eHealth interventions that are suitable for empirical examination within the mental and behavioral health domains. We view this paper as a first step in a process aimed at establishing the minimum standards that eHealth interventions should meet prior to their empirical examination within care systems. While this list of heuristics should be viewed as a starting point, it has several potential uses.

### Principal Findings

When examining proposed eHealth interventions, these heuristics can promote better communication between vendors and care systems by turning the latter into educated consumers who can define a product’s requirements and focus on user’s needs from the health care providers’ perspective.

While our main aim was to describe general principles for evaluating eHealth interventions, we believe that by outlining what is expected of these products in the mental and behavioral health domains, we have also provided the basis for developing rating scales. Rating systems developers can thus examine whether new scales that are formed based on the heuristics articulated in this paper provide additional information about the eHealth products compatibility to the clinical context.

Finally, these principles could provide common language for discussing the general potential for eHealth interventions to succeed within the clinical context. Therefore, they open the door for academic researchers to communicate about eHealth products and to assess whether certain interventions did not succeed because of their product design. For example, if an app aimed at treating depression as a standalone strategy does not provide satisfactory outcomes, it could be because such standalone technology is not effective for treating depression or it could be that the developed eHealth intervention did not meet the basic guidelines as set out in the heuristics. Being able to discuss these matters could contribute to gaining a better understanding of what works and achieving better outcomes in innovative research projects.

### Limitations

There are several limitations of the present work that should be mentioned. First, the focus of this work was to describe principles of eHealth intervention evaluation as a viewpoint. As such, it did not include psychometric measures along with case studies in order to establish its reliability and validity. Future research should focus on being able to identify and evaluate promising eHealth interventions and on investigating whether the clinical context perspective articulated in this paper contributes to making better evaluations. Second, the principles have been generalized in order to promote understanding and discussion among evaluators and product owners, and to ensure creativity in the design process. Therefore, while these heuristics might be used to locate major flaws in eHealth intervention products based on future research on that matter, it is important to note that they are not meant to be used to identify and compare nuanced differences between products. This kind of use case will be applicable only by developing rating scales that correspond to the articulated principles. Finally, we focused on the heuristics we found to be useful for evaluating products in terms of the therapeutic process. Future research should expand on the current literature and examine whether there are more heuristics that should be used in the evaluation process of eHealth interventions.

### Conclusions

This paper presents heuristic principles for evaluating eHealth interventions targeting mental and behavioral health from the perspective of the therapeutic process. While it is difficult to evaluate the potential of technologies from this perspective, this line of work may assist in establishing guidelines for product evaluation that are unique to the behavioral and mental health domains. These guidelines could help to set the bar higher for product developers and to inform health care providers about preferred eHealth intervention designs. We hope that this work will encourage all relevant stakeholders to discuss this topic further.

## Abbreviations

BIT: behavioral intervention technology
CBT: cognitive behavioral treatment
EHI: eHealth intervention
MARS: Mobile Apps Rating Scale
MFP: MyFitnessPal

## References

[1] Aitken M, Gauntlett C (2013). Patient apps for improved healthcare from novelty to mainstream. IMS Institute for Healthcare Informatics.

[2] Catwell L, Sheikh A (2009). Evaluating eHealth interventions: the need for continuous systemic evaluation. PLoS medicine.

[3] Kumar Santosh, Nilsen Wendy J, Abernethy Amy, Atienza Audie, Patrick Kevin, Pavel Misha, Riley William T, Shar Albert, Spring Bonnie, Spruijt-Metz Donna, Hedeker Donald, Honavar Vasant, Kravitz Richard, Lefebvre R Craig, Mohr David C, Murphy Susan A, Quinn Charlene, Shusterman Vladimir, Swendeman Dallas (2013). Mobile health technology evaluation: the mHealth evidence workshop. Am J Prev Med.

[4] Mohr D, Cheung K, Schueller S, Hendricks Brown C, Duan N (2013). Continuous evaluation of evolving behavioral intervention technologies. Am J Prev Med.

[5] Mohr D, Schueller S, Montague E, Burns M, Rashidi P (2014). The behavioral intervention technology model: an integrated conceptual and technological framework for eHealth and mHealth interventions. J Med Internet Res.

[6] Hekler EB, Klasnja P, Froehlich JE, Buman MP (2013). mind the theoretical gap: interpreting, using, and developing behavioral theory in HCI research.

[7] Ritterband L, Thorndike F, Cox D, Kovatchev B, Gonder-Frederick L (2009). A behavior change model for internet interventions. Ann Behav Med.

[8] Fogg B (2009). A behavior model for persuasive design.

[9] Oinas-Kukkonen H, Harjumaa M (2009). Persuasive systems design: Key issues, process model, and system features. Communications of the Association for Information Systems.

[10] Madan A, Dubey S K (2012). Usability evaluation methods: a literature review. International Journal of Engineering Science and Technology.

[11] Nielsen J (1994). Enhancing the explanatory power of usability heuristics.

[12] Karat C (1994). A comparison of user interface evaluation methods. Paper presented at: Usability inspection methods.

[13] Kientz J A, Choe E K, Birch B, Maharaj R, Fonville A, Glasson C, Mundt J (2010). Heuristic evaluation of persuasive health technologies.

[14] Nielsen J (2005). Ten usability heuristics. website.

[15] Consumer guide for selecting software and apps for managing mental health conditions.

[16] Napoli D questia Trusted online research website.

[17] Anxiety and Depression Association of America.

[18] Stoyanov S, Hides L, Kavanagh D, Zelenko O, Tjondronegoro D, Mani M (2015). Mobile app rating scale: a new tool for assessing the quality of health mobile apps. JMIR Mhealth Uhealth.

[19] (2015). Accessed August 10.

[20] Weiner J Bloomberg School of Public Health, Johns Hopkins University website.

[21] Luxton D, McCann R, Bush N, Mishkind M, Reger G (2011). mHealth for mental health: Integrating smartphone technology in behavioral healthcare. Professional Psychology: Research and Practice.

[22] Tey N P, Lai S I (2013). Correlates of and barriers to the utilization of health services for delivery in South Asia and Sub-Saharan Africa. ScientificWorldJournal.

[23] Vora KS, Koblinsky SA, Koblinsky MA (2015). Predictors of maternal health services utilization by poor, rural women: a comparative study in Indian States of Gujarat and Tamil Nadu. Journal of Health, Population and Nutrition.

[24] Jack RH, Gulliford MC, Ferguson J, Møller H (2003). Geographical inequalities in lung cancer management and survival in South East England: evidence of variation in access to oncology services?. Br J Cancer.

[25] Jones A, Haynes R, Sauerzapf V, Crawford S, Zhao H, Forman D (2008). Travel time to hospital and treatment for breast, colon, rectum, lung, ovary and prostate cancer. Eur J Cancer.

[26] Yao J, Murray AT, Agadjanian V, Hayford SR (2012). Geographic influences on sexual and reproductive health service utilization in rural Mozambique. Appl Geogr.

[27] Hoge C, Grossman S, Auchterlonie J, Riviere L, Milliken C, Wilk J (2014). PTSD treatment for soldiers after combat deployment: low utilization of mental health care and reasons for dropout. Psychiatr Serv.

[28] Pai A mobihealthnews website.

[29] Mohr D, Cuijpers P, Lehman K (2011). Supportive accountability: a model for providing human support to enhance adherence to eHealth interventions. J Med Internet Res.

